# Predicting global thermospheric neutral density during periods with high geomagnetic activity

**DOI:** 10.1038/s41598-023-47440-x

**Published:** 2023-11-21

**Authors:** Ehsan Forootan, Saeed Farzaneh, Mona Kosary, Claudia Borries, Timothy Kodikara, Maike Schumacher

**Affiliations:** 1 Geodesy Group, Department of Sustainability and Planning, Aalborg University, Rendburggade 14, 9000 Aalborg, Denmark; 2 School of Surveying and Geospatial Engineering, College of Engineering, University of Tehran, Tehran, PO.113654563 Iran; 3 Institute of Solar-Terrestrial Physics, German Aerospace Center (DLR), Kalkhorstweg 53, 17235 Neustrelitz, Germany

**Keywords:** Astronomy and planetary science, Mathematics and computing, Physics

## Abstract

Estimating global and multi-level Thermosphere Neutral Density (TND) is important for studying coupling processes within the upper atmosphere, and for applications like orbit prediction. Models are applied for predicting TND changes, however, their performance can be improved by accounting for the simplicity of model structure and the sampling limitations of model inputs. In this study, a simultaneous Calibration and Data Assimilation (C/DA) algorithm is applied to integrate freely available CHAMP, GRACE, and Swarm derived TND measurements into the NRLMSISE-00 model. The improved model, called ‘C/DA-NRLMSISE-00’, and its outputs fit to these measured TNDs, are used to produce global TND fields at arbitrary altitudes (with the same vertical coverage as the NRLMSISE-00). Seven periods, between 2003-2020 that are associated with relatively high geomagnetic activity selected to investigate these fields, within which available models represent difficulties to provide reasonable TND estimates. Independent validations are performed with along-track TNDs that were not used within the C/DA framework, as well as with the outputs of other models such as the Jacchia-Bowman 2008 and the High Accuracy Satellite Drag Model. The numerical results indicate an average 52%, 50%, 56%, 25%, 47%, 54%, and 63% improvement in the Root Mean Squared Errors of the short term TND forecasts of C/DA-NRLMSISE00 compared to the along-track TND estimates of GRACE (2003, altitude 490 km), GRACE (2004, altitude 486 km), CHAMP (2008, altitude 343 km), GOCE (2010, altitude 270 km), Swarm-B (2015, altitude 520 km), Swarm-B (2017, altitude 514 km), and Swarm-B (2020, altitude 512 km), respectively.

## Introduction

Space weather describes physical processes caused by the Sun’s radiation of energy. The manifestations of space weather are multiple, e.g., the variations of the Earth’s magnetic field or the changing states of the upper atmosphere - between the altitude of around 100 km up to 2000 km - comprising both the thermosphere and the ionosphere. This region exhibits a dynamically coupled non-linear system of chemical and physical processes.

An accurate estimation of Thermospheric Neutral Density (TND) is important for designing Low-Earth-Orbit (LEO) missions mainly those with the altitude of less than 1000 km. It is also essential, for example, to predict satellite missions’ life time, planning their required on-board fuel, performing reliable attitude control, designing orbital manoeuvre, as well as predicting and performing Earth re-entry, see discussions in, e.g.,^1–3^.

Predicting the thermosphere-ionosphere system is challenging because it is highly influenced by the solar irradiance, and it depends on the state of neutral thermospheric temperature and neutral density composition. External forces such as those related to the space weather events, e.g.,^4–6^, as well as interactions between neutral molecules with charged particles considerably influence the thermopsheric variability^7^. The Earth’s interior and surface activities, such as volcano, earthquake, or hurricane, can also change the distribution of thermosphere-ionosphere system, see, e.g.,^8^.

Empirical thermosphere or coupled thermosphere-ionosphere models are common tools to provide an estimation of TND for drag computations, however, some factors can affect their accuracy such as the simplification of model structure, coarse sampling of model inputs, and the model’s dependencies on the calibration period.

The atmospheric drag is known as a significant (non-gravitational) force that decelerates the movement of LEO satellites. Especially, LEO geodetic missions such as the Challenging Minisatellite Payload (CHAMP, 2000–2010^9^), the Gravity Recovery and Climate Experiment (GRACE, 2002–2017^10^) and its Follow-On mission (GRACE-FO, launched in 2018^11^), Gravity field and steady-state Ocean Circulation Explorer (GOCE, 2009–2013^12^), and the European Space Agency (ESA)’s Swarm mission (Swarm-A, -B, and -C launched in 2013^13^) are equipped with accelerometer sensors to measure non-gravitational forces. These measurements, after some treatments, can be used to estimate TNDs along-track of satellites with very high temporal rates (e.g., at the rate of 10 s). In the recent past, great attempts have been taken to produce these estimates from space missions such as CHAMP, GRACE, GOCE, GRACE-FO, and Swarm on-board accelerometer measurements (or from their dynamic orbits).

Various data providers, e.g., the European Space Agency (ESA, https://earth.esa.int, and research centers, e.g.,^14^, ftp://thermosphere.tudelft.nl/, and^15^ freely share their TND estimates. However, these measurements are only available along the orbits of these space missions. They might contain data gaps, and do not cover the entire globe. Therefore, it is not so easy to use them in applications such as orbit prediction, or for the global assessment of the upper atmosphere.

The along-track TND estimates have been used in previous studies to produce correction fields for thermospheric model outputs. The improved TND estimates are then considered as reanalysis or now-casting of the thermospheric variability^16–22^. To enhance the forecasting of TND fields, studies such as^23–27^ applied statistical decomposition techniques to extract dominant TND patterns. Then, state-space techniques such as the Kalman Filter (KF^28^) are applied to forecast thermospheric variations. This approach is found to be effective during geomagnetic storms because the pronounced temporal and spatial changes during these events enhance the model-data integration. An application of this technique during calm periods might be challenging because a comparable level of uncertainties in models and data reduces the efficiency of the decomposition techniques^29^.

Sequential Data Assimilation (DA) techniques are found to be efficient for merging observations and model outputs, while decreasing model uncertainties, e.g.^30–36^. The DA techniques mostly focus on updating the model states that are collocated with the along-track TND estimates. Therefore, implementing an extrapolation strategy is necessary to cover the whole globe and various altitudes. Besides, the efficiency of DA techniques during the forecasting phase depends on the initial states and how well the physical processes are represented by model equations. To take advantage of observations for modifying model structure, the Calibration and Data Assimilation (C/DA) approach was applied in^37,38^ to update the model’s states (similar to DA) and simultaneously calibrate some selected key model parameters. The calibrated parameters can then be used to simulate TNDs globally (i.e., now-cast them globally), or to forecast them in future. Therefore, no extrapolation strategy is required to extend the TND fields.

The C/DA methodology was recently applied in^38^ to re-calibrate the commonly used NRLMSISE-00 empirical thermosphere model^39^ against the TND estimates of GRACE (at the altitude of $\sim$ 410 km during February 2015). The resulting re-calibrated model, known as “C/DA-NRLMSISE-00”, was then used for now-casting TNDs and individual neutral mass compositions for 3 hours, as well as for forecasting the next 21 hours. The assessment was performed against TND estimations from the Precise Orbit Determination (POD) analysis of Swarm at the altitude range of 470–520 km during February 2–28, 2015. The geomagnetic index $K_p$ and the solar activity index $F_{107}$ of this period were varying between 2 – 5 and 110 – 150 sfu, respectively. Assessing the forecasts of TNDs with those along the Swarm-A ($\sim 467$ km), Swarm-B ($\sim 521$ km), and Swarm-C ($\sim 467$ km) orbits showed that, with respect to these missions, the Root Mean Squared Error (RMSE) was considerably reduced by 51, 57, and 54%, respectively. The authors also found a positive feedback of the new global multi-level TND fields for forecasting ionospheric variables such the electron density (*Ne*).

In response to the “ESA Swarm DISC programme 2021’s Open Call for Ideas for New data products, tools and services for Swarm”, Forootan^40^ proposed to use the C/DA approach of^38^ to leverage the publicly available CHAMP, Swarm, and GRACE derived along-track TND data, and producing a global multi-level TND product. Therefore, unlike other available global DA derived TND outputs, this multi-level estimate is purely based on openly available models and measurements. This setting makes the approach and TND results reproducible. Besides, the Swarm and GRACE(-FO) measurements provide high temporal resolution (of $\sim 10$ s), as well as dense spatial resolution in the latitudinal (north/south) direction, which can be tested whether they result in producing more accurate global TND estimates.

In this article, we present the results of^40^ by assessing the potential of the C/DA approach^37,38^ to produce multi-level global thermosphere data products consistent with Swarm and GRACE(-FO). For this,

1. seven storm periods during 2003-2022 are defined to produce global multi-level TND fields, where the along-track TND estimates of at least two satellite missions of CHAMP, GRACE, and Swarm were available;
2. along-track comparisons (validations) are performed with models such as the High Accuracy Satellite Drag Model (HASDM^41^), JB08 (^42^), and the original NRLMSISE00, as well as TNDs from CHAMP, GRACE, and Swarm during the selected seven storm periods. These data were not used during fitting the C/DA.
3. a global assessment of spatial and temporal TND changes is done by computing mean and time-variable TND biases on different altitude levels.

It is worth mentioning here that producing multi-level TND products during calm periods is not considered in this study, because, this is well covered by previous assessments, e.g., in^37,38^. The selected storm periods are associated with various of ranges of geomagnetic ($K_p$) activity (see Table 1) to make sure that the performance of the proposed technique does not depend on a certain environmental setting.

## Estimating global multi-level TND data by implementing the C/DA of NRLMSISE-00 using along-track TND as observation

The C/DA technique, as in^43^ and^37^, is a sequential approach that uses measurements to update a model’s states and simultaneously its selected model parameters. The C/DA approach is applied in this study to tune NRLMSISE-00 as basis (or background model) using along-track TND measurements such as those of CHAMP, GRACE, and Swarm. We did not use the latest version of empirical model NRLMSIS2.0 because no notable differences were found with the previous version at altitudes higher than 200 km. The implementation of C/DA is realised through a model-state equation, where the model derived TNDs and some model parameters are considered as unknowns of this system. A solution for this system is computed sequentially through minimizing the following cost function:

1 $$\begin{aligned} J({\textbf {X}})= \frac{1}{2}[{\textbf {X}}-\bar{{\textbf {X}}}^{b}]^{T} ({\textbf {P}}^{b})^{-1} [{\textbf {X}}-\bar{{\textbf {X}}}^{b}] + \frac{1}{2}[{\textbf {H}}{} {\textbf {X}}^{b}-{\textbf {Y}}]^{T} {\textbf {R}}^{-1} ({\textbf {H}}{} {\textbf {X}}^{b}-{\textbf {Y}}), \end{aligned}$$

where ${\textbf {X}}^b$ represents the ensemble of model parameters and model states, ${\textbf {P}}^{b}$ and ${\textbf {H}}$ are the error covariance matrix of the background model and the design matrix that relates TNDs to model states and parameters, respectively. The ensemble of TND measurements is represented by ${\textbf {Y}}$, and ${\textbf {R}}$ holds the uncertainty of these measurements. The details of these variables are described in what follows.

To decide which model parameters must be updated (or calibrated) within the model-state equation (Eq. (1)), we relied on our previous assessments in^37^. They showed that two model coefficients that control the density and temperature of the thermosphere, and two constants that account for the biases of the solar activity index *F*10.7 and the geomagnetic activity *Ap* are the most sensitive parameters for simulating TND changes. Therefore, this study focuses on calibrating these four key parameters within the C/DA that uses along-track TND measurements as observation for tuning the NRLMSISE-00 model. It is worth mentioning that previous studies, e.g.,^44^ indicated that indices such as *P*10.7 might be more representative of the thermosphere-ionosphere variations, or the storm-related thermospheric mass density variations could be better described by the solar wind merging electric fields, see, e.g.,^45,46^. However, these alternative indices are not considered in this study to keep the original setup of NRLMSISE-00.

First, let us assume that the original NRLMSISE-00 model is mathematically represented as:

2 $$\begin{aligned} Original\; model: F(\Theta )=F(\Theta _P,\Theta _R,\Theta _I), \end{aligned}$$

where ${\Theta }$ is a vector of parameters and input values in the model. In our formulation, we consider that ${\Theta }$ consists of ${{\Theta }_P}_{m_1\times 1}$ that are the four key parameters ($m1=4$) to be updated through the C/DA, ${{\Theta }_R}$ represents those parameters that will remain unchanged during the calibration, and ${{\Theta }_I}$ indicates the input variables such as the solar and geomagnetic indices, location, and time.

The core of C/DA is selected to be the Ensemble Kalman Filter (EnKF) as in^47^. C/DA uses the available measurements sequentially and based on their error covariance and those of model, it decides how to update the model states and its parameters. Ensembles of the model’s key parameters are generated by a Monte Carlo simulation that considers i-th (i.e., $i=1,...n$) ensemble members of the key parameters (${{\textbf {X}}}^{b}_{1,i}$) are expressed as:

3 $$\begin{aligned} {\textbf {X}}^{b}_{1,i}=\Theta _{P}+\xi _{i},\; i=1,...n, \end{aligned}$$

where ${\Theta _P}_{m_1\times 1}$ is a vector of default values of the key parameters in NRLMSISE-00 as in Eq. (2) plus random errors ($\xi _{i}$) that perturb these initial values. The standard deviations value of the Gaussian noise is considered to be 10% of each variable. In the C/DA procedure, ensembles of 90 members ($n=90$) are used to perform the numerical integration. The assimilation window is selected to be 15 minutes to three hours, and the Root Mean Squared Error (RMSE) between C/DA-NRLMSISE-00 and observed TNDs in the forecasting mode is examined to derive the suitable length.

The last set of key parameters that are estimated in the sequential C/DA are considered as the optimal calibrated parameter set, which provides us with ${\hat{\Theta }}_{P}$. These parameters then replace the default values of the original NRLMSISE-00 model in Eq. (2) to now-cast and forecast (for the next hour) multi-level TNDs, individual neutral mass densities, and thermospheric temperature globally. Details of this implementation is documented in^38^. The C/DA model, i.e., called ‘C/DA-NRLMSISE-00’, is represented by:

4 $$\begin{aligned} C/DA\; model: F({{\hat{\Theta }}}_P,\Theta _R,\Theta _I), \end{aligned}$$

which is used for computing the global multi-level TND data of this study. Here, we chose the forecast fields of the C/DA-NRLMSISE-00 for our investigations because their uncertainty is generally higher than the analysis period (where the along-track data is used to fit the C/DA model). Therefore, if the forecast fields satisfy the quality measures, it is likely the analysis fields do the same. Another motivation to select the forecast fields to be investigated is that they can be used for studies that require predictions of TND in future (some period ahead of the available along-track data), for example, in orbit propagation and orbit prediction applications.

Selecting realistic spatial and temporal resolution to produce the global multi-level TND data is discussed in^40^, where a spatial resolution of about five degrees, a vertical resolution of 25 km, and a temporal resolution of 1 hours are found to be realistic.

To numerically evaluate the performance of models and the multi-level data, compared to observations, the statistical measures such as bias, relative error, RMSE, improvement percentage, Average of Absolute Percentage Deviation (AAPD), fit, Coefficient Of Efficiency (COF), and Correlation Coefficients (CC) are applied that are introduced in^40^.

## Results

### Selecting the storm periods

**Figure 1:**
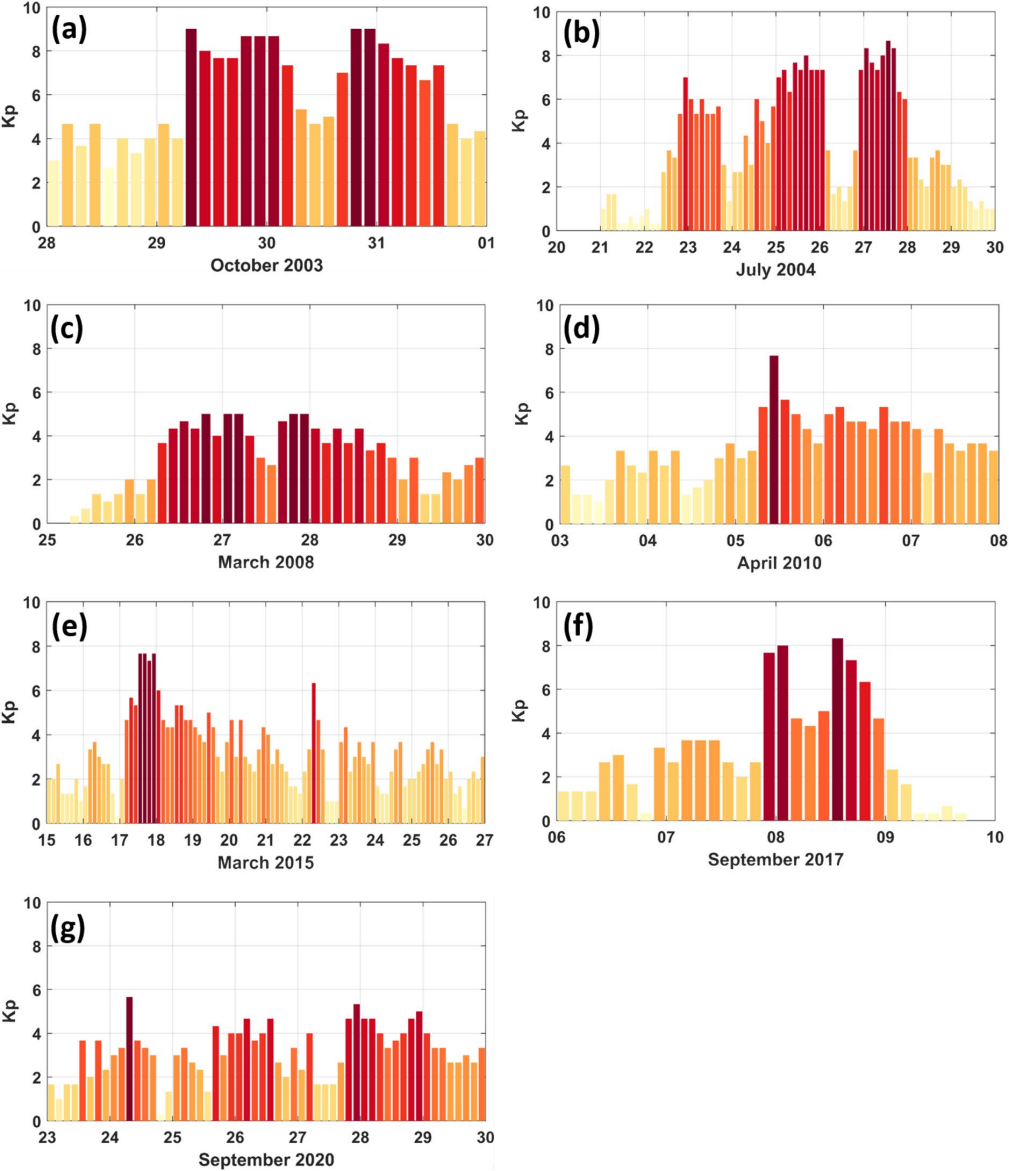
An overview of the geomagnetic activity, represented by the (*Kp*) index during the seven periods (between 2003–2020) selected in this study. The indices are downloaded from https://kp.gfz-potsdam.de/en/. The dates of selected storms, as well as the average and the range of $K_p$ in these storms are reported in Table 1.

The CHAMP, GRACE, GOCE, and Swarm space missions cover the years of 2000-2010, 2002-2017, 2009-2013, and 2013-now, respectively. The measurements of these missions are used within the C/DA to study the feasibility of along-track measurements for generating global multi-level TND fields. For our investigations, periods with considerable geomagnetic activity, i.e., with considerable $K_p$ fluctuations, are extracted (see Table 1). Seven periods are chosen based on the coverage of these missions, where they are among strong geomagnetic storm periods during 2003–2020. The corresponding geomagnetic changes are shown in Figure 1, while the dates of these storms are listed in Table 1.

**Table 1:** An overview of the along-track TND measurements used as observation within the C/DA to produce the global multi-level TND products, and those that we used for validation. Dates of the assessed storms, as well as average and range of the $K_p$ index are reported.

Storm ID	Date	Assimilation (Altitude km)	Validation (Altitude km)	Average $K_p$	Range of $K_p$
Storm1	2003/10/28-31	CHAMP (401.13)	GRACE (490.91)	6	4–8
Storm2	2004/07/21-29	CHAMP (386.60)	GRACE (486.34)	4	1–8
Storm3	2008/03/25-29	GRACE (477.74)	CHAMP (343.75)	3	1–5
Storm4	2010/04/03-07	CHAMP (301.59)	GOCE (270.03)	4	1–8
Storm5	2015/03/15-26	Swarm-C (466.86)	Swarm-B (520.69)	3	2–8
Storm6	2017/09/06-09	Swarm-C (451.35)	Swarm-B (514.80)	3	1–8
Storm7	2020/09/23-29	Swarm-C (444.48)	Swarm-B (512.72)	3	2–6

### Along-track investigation of the TND estimates during the seven storm events

**Figure 2:**
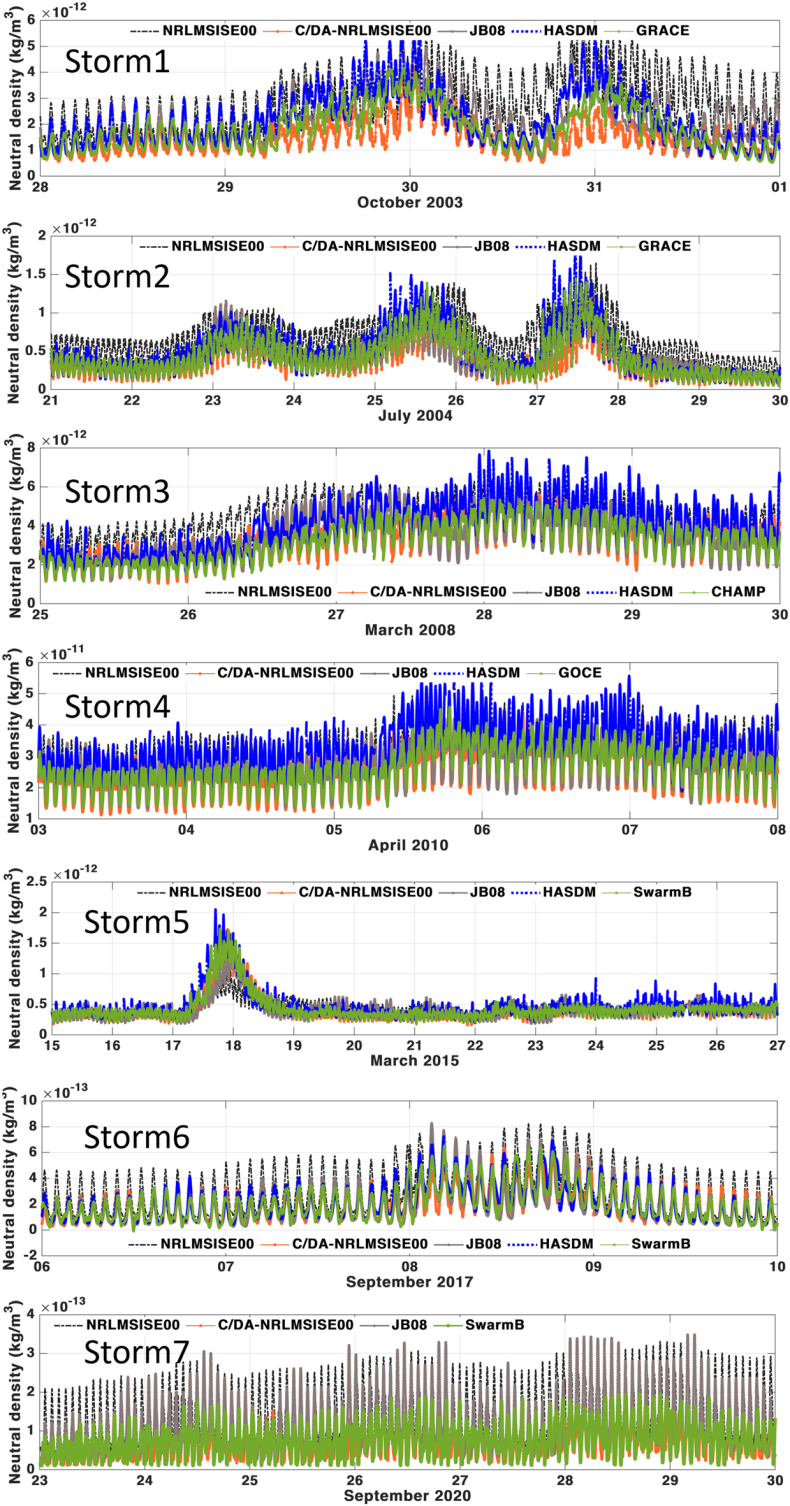
A comparison between the forecasts of C/DA-NRLMSISE-00 and those of the original NRLMSISE-00 model, as well as JB08, HASDM models along-track of the satellites that were not used to estimate the multi-level data. The C/DA results are in the forecast mode. The altitudes of these satellite missions, used for validation, are presented in Table 1.

In what follows, the outputs derived from C/DA-NRLMSISE-00 (used for generating the multi-level TND products) are investigated during Storm1-Storm7. The satellite mission data used for performing the C/DA and validation are summarised in Table 1. We also compared the TND estimates of C/DA with those of the original NRLMSISE-00 model, as well as JB08 and HASDM models. The C/DA results are presented in the forecasting mode, which means that the C/DA-NRLMSISE-00 (Eq. (4)) has already been preformed using the TND data of three hours before and the satellite derived TNDs that we show in Figure 2 are not used within the C/DA. The results along-track of the missions that are used for the C/DA are shown in the Supplementary file related to this article. In this section, we only show the validation against another satellite measurements that are not at the same altitude. It means that the results are validated at horizontal locations and altitudes that are different from the along-track TNDs used as observation within the C/DA.

The complete statistical investigations are presented in Table 2. In all the investigated periods, estimates of the C/DA-NRLMSISE-00 are found to be closer to the independent along-track estimations compared to the original NRLMSISE-00, JB08, and HASDM. There is an exception during storm 2, where HASDM performs slightly better (RE of 44.64% for HASDM compared to 50.51% for the C/DA). In terms of RMSE, however, the biases of the two models are found to be close during this storm. Thus, the numerical differences can be considered as statistically insignificant. Considering the value of biases, our results indicate that the original NRLMSISE-00 and JB08 exhibit considerable biases, where the first indicate overestimation in most of the storm events and the latter in those after 2008 (Storm3 to Storm7). They both indicate difficulties in catching the peak of the storms and shows different responses to the storm, i.e., different storm duration is observed from the models to return to the TND magnitude of the calm period.

The magnitude of biases is found to be in the range of the TND signal in those altitudes, e.g., $10^{-12}$ for the height of $\sim$250–400 km. This error magnitude cannot be ignored in precise orbit prediction applications. Application of the C/DA against available along-track TND estimates reduce the biases to one level magnitude smaller, which is in the range of the noise of TND estimates. By performing various along-track comparisons, we observed that the magnitude of TND estimates from GRACE measurements is often relatively bigger than that of other missions. For example, if one takes CHAMP estimates and transfer the TNDs to the altitude of GRACE (e.g., using a vertical exponential conversion, as in^21,37^), it can be seen that the amplitude of GRACE TNDs is relatively bigger than those of the transformation. Besides, by comparing (1) other model outputs, (2) those of satellite missions, and (3) the C/DA, we noted that the TND amplitudes of other models overestimate the magnitude of TNDs during the assessed events. Those of C/DA (in 3) are close to those of satellites (in 2) or slightly smaller. In our view, this can be because of that fact that: (i) the C/DA is tuned to the satellite derived TNDs, therefore, their closeness is expected; and (ii) the C/DA results are in the forecast mode, while those of other models are in the analysis mode. It is often the case that the forecast mode is relatively smooth. Nevertheless, the C/DA results are found to have acceptable accuracy, compared to the satellite estimate, and therefore, this approach is selected to produce the global and multi-level TND data.

**Table 2:** A summary of statistical measures between the NRLMSISE-00, JB08, HASDM, and the TND forecasts of C/DA-NRLMSISE-00 compared to the TND estimates of CHAMP, GRACE, GOCE and Swarm during the seven storm periods of Table 1.

Storm1	October 2003	Validation: GRACE	Altitude: 490.91 km	Mode: Forecast	1h forecast	RE(%)
Model	RMSE (kg/$m^3$)	Bias (kg/$m^3$)	Coefficient of efficiency	Correlation	AAPD (%)	
NRLMSISE-00	1.28$\times 10^{-12}$	1.03$\times 10^{-12}$	−1.43	0.54	71.15	57.91
JB08	6.74$\times 10^{-13}$	3.47$\times 10^{-13}$	0.32	0.68	32.35	30.49
HASDM	6.75$\times 10^{-13}$	3.56$\times 10^{-13}$	0.32	0.66	28.06	29.76
C/DA-NRLMSISE-00	6.16$\times 10^{-13}$	-3.34$\times 10^{-13}$	0.43	1.03	22.46	27.84

Storm2	July 2004	Validation: GRACE	Altitude: 486.34 km	Mode: Forecast	1h Forecast	RE(%)
Model	RMSE (kg/$m^3$)	Bias (kg/$m^3$)	Coefficient Of Efficiency	Correlation	AAPD (%)	
NRLMSISE-00	2.89$\times 10^{-13}$	2.55$\times 10^{-13}$	−0.33	0.80	79.94	82.20
JB08	1.37$\times 10^{-13}$	1.31$\times 10^{-14}$	0.69	0.89	24.47	39.12
HASDM	1.59$\times 10^{-13}$	6.77$\times 10^{-14}$	0.58	0.70	25.28	44.64
C/DA-NRLMSISE-00	1.42$\times 10^{-13}$	-6.65$\times 10^{-14}$	0.67	1.03	20.28	50.51

Storm3	March 2008	Validation: CHAMP	Altitude: 343.75 km	Mode: Forecast	1h Forecast	RE(%)
Model	RMSE (kg/$m^3$)	Bias (kg/$m^3$)	Coefficient Of Efficiency	Correlation	AAPD (%)	
NRLMSISE-00	1.21$\times 10^{-12}$	1.01$\times 10^{-12}$	−0.46	0.73	37.93	45.66
JB08	6.97$\times 10^{-13}$	2.40$\times 10^{-13}$	0.51	0.72	17.82	26.25
HASDM	1.09$\times 10^{-12}$	8.20$\times 10^{-13}$	−0.19	0.63	28.38	40.24
C/DA-NRLMSISE-00	5.33$\times 10^{-13}$	1.89$\times 10^{-13}$	0.71	0.84	14.84	20.08

Storm4	April 2010	Validation: GOCE	Altitude: 270.03 km	Mode: Forecast	1h Forecast	RE(%)
Model	RMSE (kg/$m^3$)	Bias (kg/$m^3$)	Coefficient of efficiency	Correlation	AAPD (%)	
NRLMSISE-00	5.77$\times 10^{-12}$	2.90$\times 10^{-12}$	0.14	0.63	18.60	46.25
JB08	5.40$\times 10^{-12}$	2.01$\times 10^{-12}$	0.24	0.64	17.42	43.32
HASDM	8.35$\times 10^{-12}$	6.51$\times 10^{-12}$	−0.80	0.58	27.59	65.51
C/DA-NRLMSISE-00	4.29$\times 10^{-12}$	-6.77$\times 10^{-13}$	0.52	0.71	12.72	34.41

Storm5	March 2015	Validation: Swarm-B	Altitude: 520.69 km	Mode: Forecast	1h Forecast	RE(%)
Model	RMSE (kg/$m^3$)	Bias (kg/$m^3$)	Coefficient of efficiency	Correlation	AAPD (%)	
NRLMSISE-00	1.41$\times 10^{-13}$	3.02$\times 10^{-15}$	0.57	1.48	18.02	60.95
JB08	1.19$\times 10^{-13}$	-1.86$\times 10^{-14}$	0.69	1.05	18.54	51.55
HASDM	1.15$\times 10^{-13}$	3.66$\times 10^{-14}$	0.71	0.80	20.08	48.81
C/DA-NRLMSISE-00	7.48$\times 10^{-14}$	-1.61$\times 10^{-14}$	0.87	0.97	10.46	32.31

Storm6	September 2017	Validation: Swarm-B	Altitude: 514.80 km	Mode: Forecast	1h Forecast	RE(%)
Model	RMSE (kg/$m^3$)	Bias (kg/$m^3$)	Coefficient of efficiency	Correlation	AAPD (%)	
NRLMSISE-00	1.27$\times 10^{-13}$	1.01$\times 10^{-13}$	0.07	0.68	147.18	67.83
JB08	6.58$\times 10^{-14}$	-1.13$\times 10^{-14}$	0.75	0.81	65.02	34.96
HASDM	6.09$\times 10^{-14}$	9.41$\times 10^{-15}$	0.78	0.85	76.74	32.16
C/DA-NRLMSISE-00	5.78$\times 10^{-14}$	-3.99$\times 10^{-15}$	0.80	0.91	70.63	30.76

Storm7	September 2020	Validation: Swarm-B	Altitude: 512.72 km	Mode: Forecast	1h Forecast	RE(%)
Model	RMSE (kg/$m^3$)	Bias (kg/$m^3$)	Coefficient of efficiency	Correlation	AAPD (%)	
NRLMSISE-00	8.36$\times 10^{-14}$	6.58$\times 10^{-14}$	−3.29	0.40	110.29	141.23
JB08	5.97$\times 10^{-14}$	3.16$\times 10^{-14}$	−1.18	0.41	67.15	100.86
C/DA-NRLMSISE-00	3.05$\times 10^{-14}$	-8.50$\times 10^{-15}$	0.42	0.73	39.40	51.52

### A global assessment of the multi-level TND data

To illustrate what could be expected as a global impact of integrating along-track TNDs with NRLMSISE-00 during a period with high and moderate geomagnetic activity, the Principal Component Analysis, PCA^48^, is applied to the data of the all seven storms. However, the results during Storm5 and Storm7 are shown here as an example. For this, the TND estimates of the original and the global multi-level data are considered globally with 30 minutes temporal sampling at 350 km altitude (the altitude is chosen to be in the range of the satellite missions used in this study). The differences are then computed with the corresponding estimations of NRLMSISE-00. Finally, PCA is applied on the global half-hourly differences of TNDs at 350 km altitude. More comparisons can be found in^40^.

**Figure 3:**
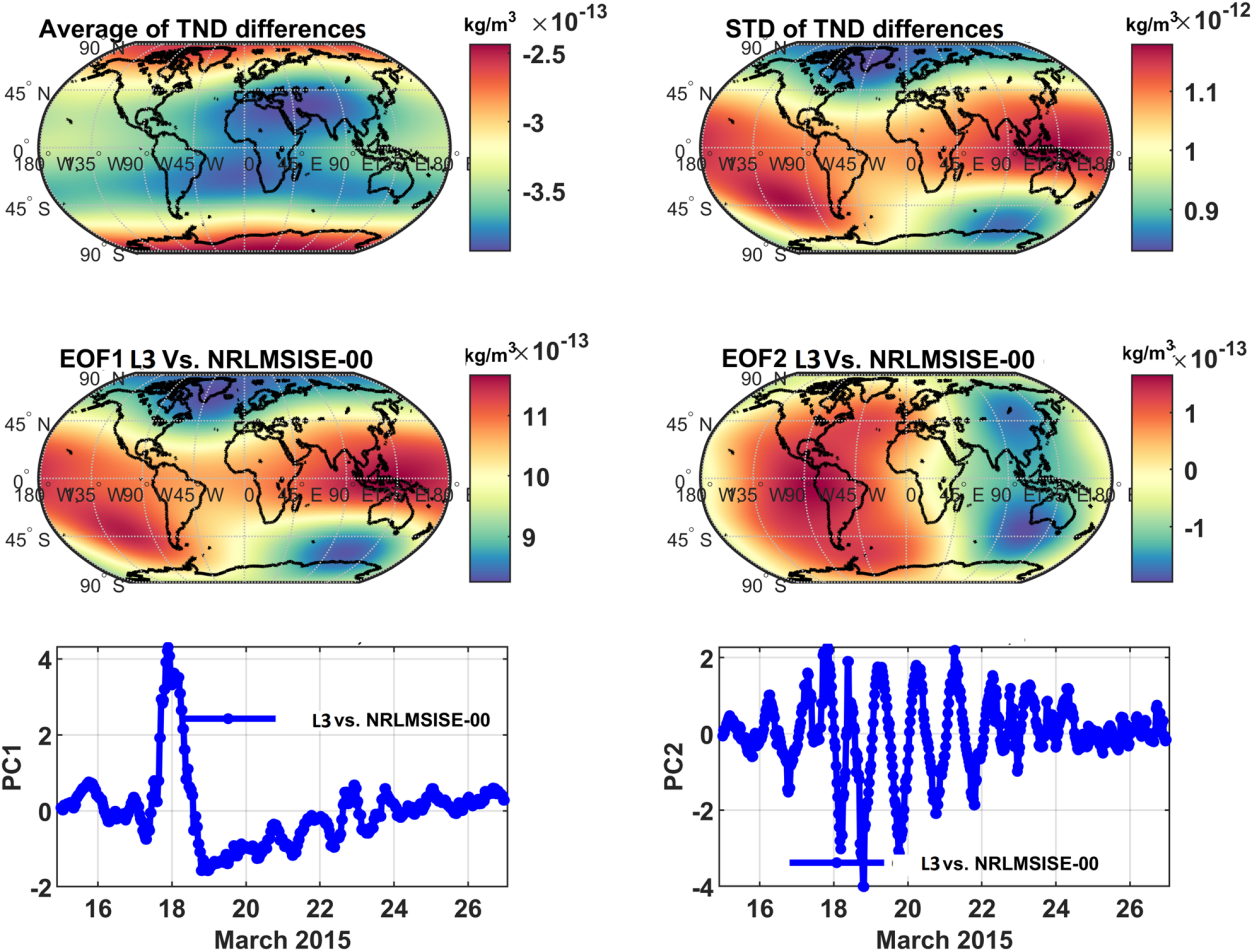
PCA of the TND differences between NRLMSISE-00 and C/DA-NRLMSISE-00 at 350 km during March 2015. The anomaly maps (EOFs) are in terms of kg/m^3^, which can be multiplied by the unit less time series (PCs) on the right to derive orthogonal modes. The first mode of differences represents 97% of the total variance of TND differences and the second mode indicates 0.89% of the variance.

**Figure 4:**
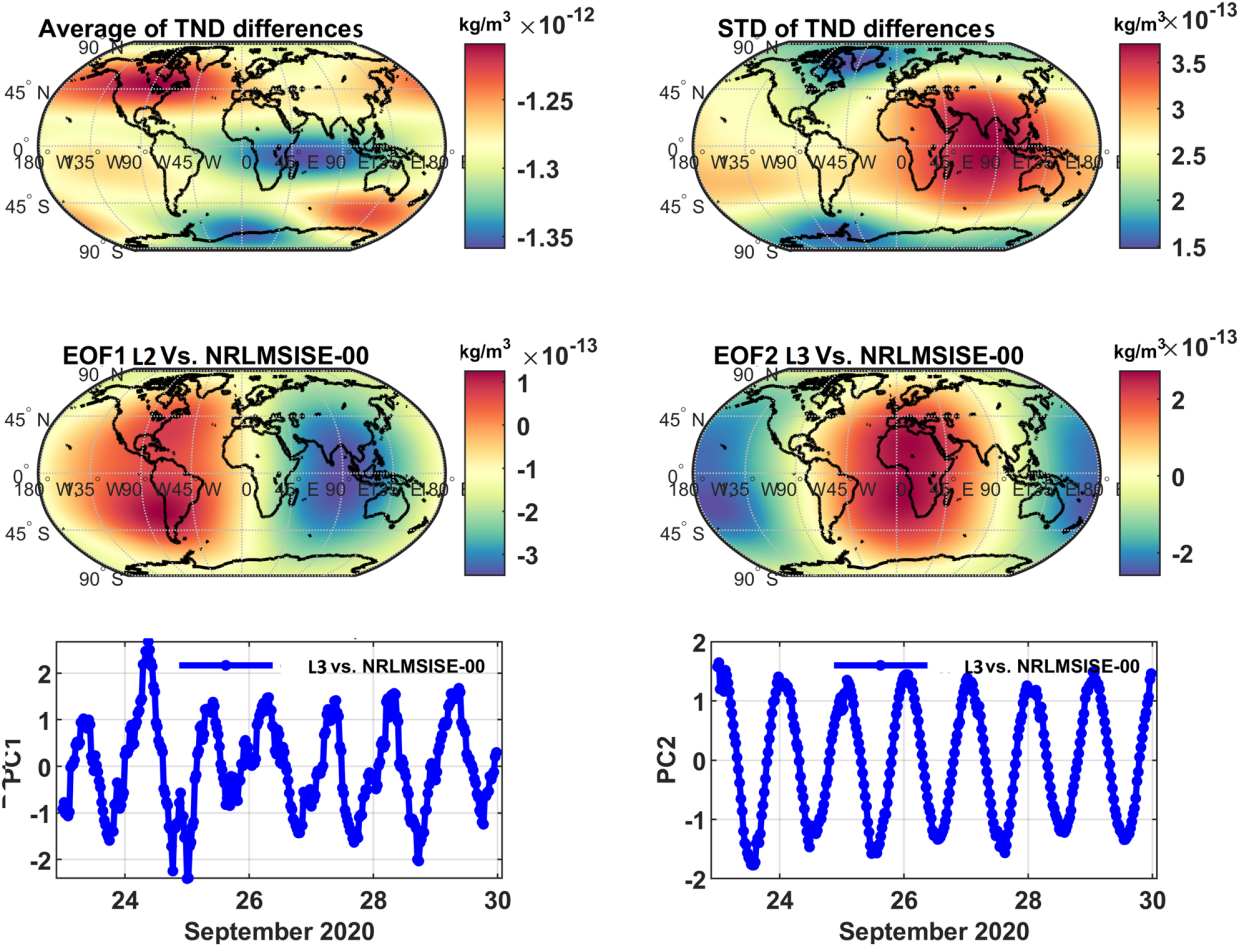
An overview of the PCA results derived from the global TND differences between the original NRLMSISE-00 and C/DA-NRLMSISE-00 at 350 km during Storm7 in September 2020. The anomaly maps (EOFs) are in terms of kg/$m^3$, which can be multiplied by the unit less time series (PCs) on the right to derive orthogonal modes. The first mode of differences represents 42% of the total variance of TND differences and the second mode indicates 29% of the variance.

The average and standard deviations of the global TNDs, as well as the first two dominant PCA modes are shown in Figs. 3 and 4. Summaries of the differences for the altitude of 350 km are reported in Table 3 , which indicate that the errors are considerable, and therefore, they are considerable for orbit prediction applications.

**Table 3:** An overview of the global errors in estimating TND. Statistics are estimated as differences between the TNDs of C/DA and the original NRLMSISE-00. The TND values are reported in kg/m^3^. Standard deviations is shown by ‘Std’.

Altitude (km)	Bias Year 2015	Bias Year 2020	Std Year 2015	Std Year 2020
350	$\sim 2.5\times 10^{-13}$	$\sim 1.3\times 10^{-12}$	$\sim 1.05\times 10^{-12}$	$\sim 3.5\times 10^{-13}$

The first two modes (mode1: EOF1 and PC1 as well as mode 2: EOF2 and PC2) of the TND differences (between the multi-level data and the original NRLMSISE-00 model outputs) indicate the main differences of the two products correspond to the representation of TND changes due to the geomagnetic storm and the diurnal density fluctuations. The impact of the storm is better demonstrated in 2015, where the magnitude of the geomagnetic activity changed considerably, see EOF1 and PC1 of Figure 3. maximum magnitude of the first and the second mode are found to be $5\times 10^{-12}$ kg/m^3^ and $0.37\times 10^{-12}$ kg/m^3^, respectively. Here, the magnitude is computed by multiplying the maximum value of EOFs with the maximum value of PC.

The average magnitude of the first two modes of the differences in Figure 4 are found to be around $10^{-13}$ kg/m^3^, where in the first mode (EOF1 and PC1) indicates a mixture of the diurnal/semi-diurnal differences, and a jump due to the geomagnetic changes on September 25-26 can be detected (see the plots on middle- and bottom-left). The second mode (EOF2 and PC2) is dominated by the out of phase diurnal differences between the two models (see the plots on middle- and bottom-right).

**Figure 5:**
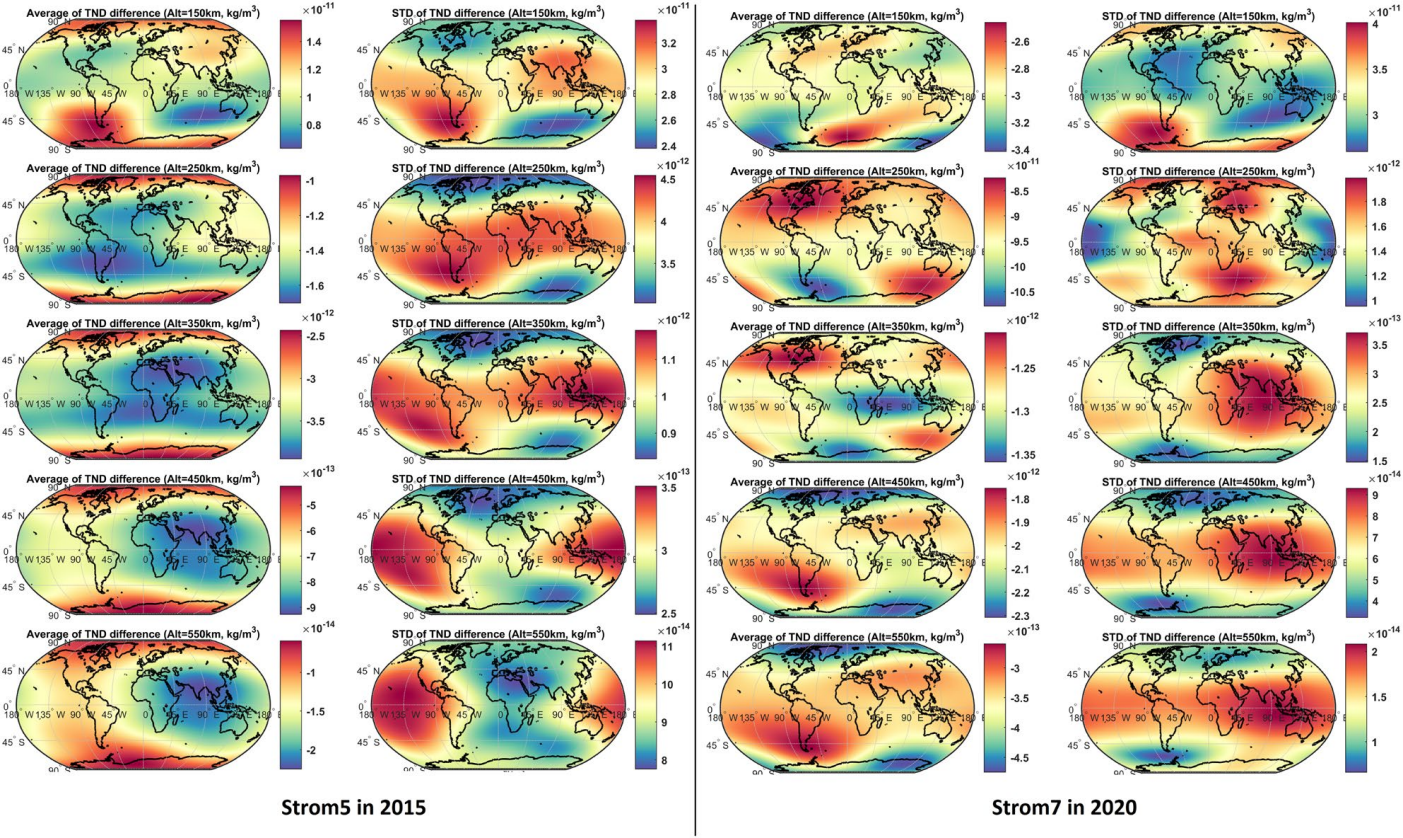
An overview of the mean and standard deviation of the global TND differences between the C/DA-NRLMSISE-00 (multi-level TND data) and original NRLMSISE-00. The results are presented for the altitudes of 150, 250, 350, 450, and 550 km during Storm5 in March 2015 and Storm7 in September 2020.

In order to understand the effect of the new products on the estimation of the global TND changes on different altitudes, we computed half-hourly products using the C/DA-NRLMSISE-00 (for producing the multi-level data) and the original NRLMSISE-00 models. The temporal average and the standard deviations of their differences are shown in Figure 5 and the statistics are reported in Table 4, where the results are related to the vertical altitudes of 150, 250, 350, 450, and 550 km during Storm5 in 2015 and Storm7 in 2020. The magnitude of differences at different altitudes is found considerable, where biases can directly affect the estimation of drag coefficients, and the standard deviations can contribute to accumulative errors, for example, in orbit prediction applications.

**Table 4:** An overview of the global errors in estimating TND. Statistics are estimated as differences between the TNDs of C/DA and the original NRLMSISE-00. The TND values are reported in kg/m^3^. Standard deviations is shown by ‘Std’.

Altitude (km)	Bias year 2015	Bias year 2020	Std year 2015	Std year 2020
150	~1.2 × 10^−11^	~2.9 × 10^−11^	~2.9 × 10^−11^	~3.4 × 10^−11^
250	~1.0 × 10^−11^	~8.5 × 10^−12^	~9.5 × 10^−12^	~1.4 × 10^−12^
350	~1.2 × 10^−12^	~2.5 × 10^−13^	~1.2 × 10^−12^	~2.5 × 10^−13^
450	~ 2.1 × 10^-12^	~6.5 × 10^−14^	~2.1 × 10^−12^	~6.5 × 10^−14^
550	~1.4 × 10^−14^	~3.5 × 10^−13^	~4.1 × 10^−13^	~1.5 × 10^−14^

## Conclusion

In this study, we investigated the possibility of applying the publicly available along-track TND data for producing a global multi-level TND data set. Unlike other available global data assimilation outputs, the proposed approach and the multi-level TND data product is based on openly available data, which makes it reproducible. Besides, CHAMP, Swarm and GRACE(-FO) data provide the opportunity to produce high resolution and continuous global TND estimates.

To achieve a comprehensive assessment, seven periods between 2003-2020 with considerable geomagnetic activity are considered (the periods are labeled Storm1 to Storm7 in the previous chapter, see Figure 1). During these events, various combinations of the along-track TND estimates are investigated to produce continuous, global, and multi-level TND products. The validations are performed in the (one-hour) forecast phase with the along-track data that were not used for our production. The study has tested the observations of CHAMP, GRACE and Swarm to be used for producing global multi-level TND products and those of CHAMP, GRACE, GOCE and Swarm are applied for validation, see Table 1. Having said that, it should be mentioned here that in this study, we did not investigate the long-term consistency of along-track TND estimates from different satellite missions. There have been many discussions that illustrate biases between the TNDs from CHAMP, GRACE and Swarm, see e.g.,^14,36,37^. A reliable bias elimination must be applied to produce long-term consistent global multi-level TNS products.

Our experience considers the NRLMSISE-00 model^39^ as basis and the simultaneous Calibration and Data Assimilation, C/DA^37,38^, is applied to fit the original modelled TND outputs to those of the along-track estimates. Therefore, the new model is named C/DA-NRLMSISE-00 (Eq. (4)), which can be used to simulate TNDs and individual neutral components globally on various altitudes. The updated model might be useful for applications such as orbit determination and space weather. To produce the end-user multi-level TND data, meaningful spatial and temporal sampling and the vertical sensitivity are also investigated, which are covered by^40^. In what follows, the featured conclusions are summarised.

1. The global investigations of the TND estimates, e.g., drawn by applying PCA in Section 5.3, indicate that even though the vertical coverage of along-track measurements is limited, C/DA can transfer their updates to various altitude levels. Our investigations indicate that the range of impact is the same as the vertical coverage of the basis model, i.e., NRLMSISE-00. However, our validation is limited by the data availability. For example, we could test the minimum altitude of around 270 km using GOCE during Storm4 in 2010 and the maximum altitude of around 540 km using Swarm data in Storm5, 6, and 7. More validations with available drag estimates of space objects might provide more insights about the performance of the C/DA and the new multi-level TND data products.
2. The dominant changes in the TND estimates derived from the PCA, as well as computing the biases between the original and C/DA-NRLMSISE-00 are found to be considerably big for many geodetic applications. Other available models such JB08 and HASDM also indicate biases, where that of JB08 is found to be similar to the original NRLMSISE-00, but much smaller values are found for HASDM. During Storm4 in 2010, HASDM indicates considerable bias of $\sim 10^{-12}$ kg/m^3^ at the altitude of GOCE, i.e., $\sim 270$ km. Generally speaking, a magnitude of bias around $1-4 \times 10^{-12}$ kg/m^3^ at the altitude of $\sim$ 300-400 km can be found from most of the available models, which will be considerably decreased by producing the proposed global TND data.
3. The choice of spatial (horizontal and vertical) and temporal sampling is investigated in^40^ by implementing empirical covariance matrices. The results indicate that the time interval of 45 minutes to one hour, the horizontal sampling of around five degrees, and the vertical sampling of about 25 km can be realistic for producing the final global multi-level TND fields. This investigation is however limited to the selection of the NRLMSISE-00 as the basis model and the few periods that are covered in this study. A more comprehensive assessment might provide alternative suggestions. Besides, our recommendation does not take into the account the end-user requirements and neither the data storage capacity, as well as download and upload requirements for sharing long-term multi-level TND data.
4. In this study, a short-term (1h to 21h) prediction of the TND fields is assessed (using the C/DA-NRLMSISE-00), which was found to be accurate. A long term assessment of the prediction skills in terms of forecasting the total neutral density values and the density of individual neutral elements will be helpful to understand the contribution of the C/DA approach and the new global and multi-level TND data in space weather applications.

The multi-level TND fields of this study could be further compared to available data sets, such as that of^49^, to understand to what extent they are in agreement, especially at and beyond the edges of the ranges covered by the satellite derived TNDs. This assessment will be considered in future contributions.

## Open research

The Thermosphere Neutral Density (TND) data are available from the European Space Agency (ESA) https://earth.esa.int, and Delft Technical University ftp://thermosphere.tudelft.nl/. The (*Kp*) indices are downloaded from https://kp.gfz-potsdam.de/en/. The multi-level TND data is freely available through the ESA’s Swarm DISC programme (https://swarm-diss.eo.esa.int/#swarm%2FAdvanced%2FPre-studies%2FMultilevel_global_thermosphere_data) and the Github of the Geodesy Group at Aalborg University (https://github.com/AAUGeodesy/StormNeutralDensity/tree/main) . The NRLMSISE-00 model is freely available from the US Naval Research Laboratory https://map.nrl.navy.mil/map/pub/nrl/NRLMSIS/. The full technical report of^40^ can be found from https://earth.esa.int/eogateway/activities/swarm-disc-pre-study-5-2.

### Supplementary Information

Supplementary Information.

## Data Availability

The new global multi-level TND data generated during the current study are available from https://github.com/AAUGeodesy/StormNeutralDensity/tree/main and https://swarm-diss.eo.esa.int/#swarm%2FAdvanced%2FPre-studies%2FMultilevel_global_thermosphere_data.
