# Supplementary material for: Predicting global thermospheric neutral density during periods with high geomagnetic activity
Source: Sci Rep. 2023 Nov 21;13:20322. doi: 10.1038/s41598-023-47440-x (PMC10663592; doi:10.1038/s41598-023-47440-x)
Supplement: Supplementary file 1 — Supplementary Information. [file 41598_2023_47440_MOESM1_ESM.pdf]

Supplementary to: Predicting global thermospheric neutral density during periods with high geomagnetic activity

Ehsan Forootan[1,*], Saeed Farzaneh[2], Mona Kosary[2], Claudia Borries[3], Timothy Kodikara[3], and Maike Schumacher[1]

[1]Geodesy Group, Department of Sustainability and Planning, Aalborg University, Rendburggade 14, 9000, Aalborg, Denmark
[2]School of Surveying and Geospatial Engineering, College of Engineering, University of Tehran, PO.113654563, Tehran, Iran
[3]Institute of Solar-Terrestrial Physics, German Aerospace Center (DLR), Kalkhorstweg 53, 17235, Neustrelitz, Germany

ABSTRACT

This document provides an extra investigation along-track of the satellite missions used in Forootan et al. (2023) "Predicting global and multi level thermospheric neutral density during periods with high geomagnetic activity".

Validation along-track of the satellite missions used for the C/DA

In this section, we compare the along-track C/DA-NRLMSISE-00 (multi-level TND data) results in the forecast mode. This means that the C/DA-NRLMSISE-00 has already been preformed using the TND data of three hours before and the satellite derived TNDs that we show in Figure (1) are not used within the C/DA. However, the validation observations are along the same orbit that are used in the C/DA. Our numerical results indicate that, compared to other models, the C/DA estimates are closer to the along-track measurements. The improvement is clearly seen for both Correlation Coefficient (CC) and the Relative Error (RE) measures, where the heights CCs and smallest REs are found to be associated with the global multi-level data. Table 1 summarises all the statistical results within the seven storm periods.

Table 1. A summary of statistical measures between the NRLMSISE-00, JB08, HASDM, and the TND forecasts of C/DA-NRLMSISE-00 compared to the TND estimates of CHAMP, GRACE and Swarm during the seven storm periods of Table 1 of the manuscript.

Storm1	October 2003	Assimilation: CHAMP	Altitude: 401.13 km	Mode: Forecast	1h Forecast	
Model	RMSE (kg/m^3)	Bias (kg/m^3)	Coefficient Of Efficiency	Correlation	AAPD (%)	RE(%)
NRLMSISE-00	5.08×10^{-12}	4.28×10^{-12}	-3.50	0.43	74.87	84.31
JB08	2.52×10^{-12}	1.49×10^{-12}	-0.10	0.57	31.22	41.79
HASDM	2.37×10^{-12}	1.49×10^{-12}	0.01	0.60	27.22	38.51
C/DA-NRLMSISE-00	1.74×10^{-12}	5.79×10^{-14}	0.46	0.72	21.64	28.99
Storm2	July 2004	Assimilation: CHAMP	Altitude: 386.60 km	Mode: Forecast	1h Forecast	
Model	RMSE (kg/m^3)	Bias (kg/m^3)	Coefficient Of Efficiency	Correlation	AAPD (%)	RE(%)
NRLMSISE-00	1.63×10^{-12}	1.41×10^{-12}	-0.45	0.72	68.50	100.34
JB08	0.83×10^{-12}	3.41×10^{-13}	0.61	0.79	28.08	51.63
HASDM	1.01×10^{-12}	6.30×10^{-13}	0.42	0.68	30.59	61.16
C/DA-NRLMSISE-00	0.61×10^{-12}	-9.39×10^{-14}	0.79	0.91	17.83	37.75
Storm3	March 2008	Assimilation: GRACE	Altitude: 477.74 km	Mode: Forecast	1h Forecast	
Model	RMSE (kg/m^3)	Bias (kg/m^3)	Coefficient Of Efficiency	Correlation	AAPD (%)	RE(%)
NRLMSISE-00	1.33×10^{-13}	8.90×10^{-14}	0.31	0.67	46.44	47.33
JB08	9.94×10^{-14}	1.21×10^{-14}	0.62	0.69	27.88	35.30
HASDM	9.62×10^{-14}	4.22×10^{-14}	0.64	0.73	27.75	33.59
C/DA-NRLMSISE-00	6.72×10^{-14}	-1.93×10^{-15}	0.82	0.84	21.89	23.87
Storm4	April 2010	Assimilation: CHAMP	Altitude: 301.59 km	Mode: Forecast	1h Forecast	
Model	RMSE (kg/m^3)	Bias (kg/m^3)	Coefficient Of Efficiency	Correlation	AAPD (%)	RE(%)
NRLMSISE-00	5.37×10^{-12}	2.98×10^{-12}	-3.18	0.16	43.92	58.44
JB08	5.31×10^{-12}	2.42×10^{-12}	-3.09	0.17	40.83	57.82
HASDM	6.01×10^{-12}	3.99×10^{-12}	-4.26	0.22	47.70	64.40
C/DA-NRLMSISE-00	4.02×10^{-12}	7.33×10^{-13}	-1.34	0.25	31.19	43.77
Storm5	March 2015	Assimilation: Swarm-C	Altitude: 466.86 km	Mode: Forecast	1h Forecast	
Model	RMSE (kg/m^3)	Bias (kg/m^3)	Coefficient Of Efficiency	Correlation	AAPD (%)	RE(%)
NRLMSISE-00	2.72×10^{-13}	3.66×10^{-14}	0.57	1.20	16.40	50.58
JB08	2.44×10^{-13}	7.13×10^{-15}	0.65	0.87	16.47	45.39
HASDM	2.52×10^{-13}	1.20×10^{-13}	0.63	0.75	16.86	45.85
C/DA-NRLMSISE-00	1.44×10^{-13}	-1.26×10^{-14}	0.88	0.96	8.61	26.82
Storm6	September 2017	Assimilation: Swarm-C	Altitude: 451.35 km	Mode: Forecast	1h Forecast	
Model	RMSE (kg/m^3)	Bias (kg/m^3)	Coefficient Of Efficiency	Correlation	AAPD (%)	RE(%)
NRLMSISE-00	2.88×10^{-13}	2.46×10^{-13}	0.07	0.78	54.16	54.60
JB08	1.57×10^{-13}	-3.98×10^{-14}	0.72	0.87	19.17	29.85
HASDM	1.73×10^{-13}	5.86×10^{-14}	0.66	0.74	21.28	32.33
C/DA-NRLMSISE-00	1.26×10^{-13}	-1.52×10^{-14}	0.82	0.96	16.19	23.82
Storm7	September 2020	Assimilation: Swarm-C	Altitude: 444.48 km	Mode: Forecast	1h Forecast	
Model	RMSE (kg/m^3)	Bias (kg/m^3)	Coefficient Of Efficiency	Correlation	AAPD (%)	RE(%)
NRLMSISE-00	2.45×10^{-13}	2.16×10^{-13}	-4.43	0.46	91.09	131.28
JB08	1.53×10^{-13}	1.06×10^{-13}	-1.13	0.47	48.48	82.36
C/DA-NRLMSISE-00	0.71×10^{-13}	-3.75×10^{-15}	0.54	0.71	21.35	37.88

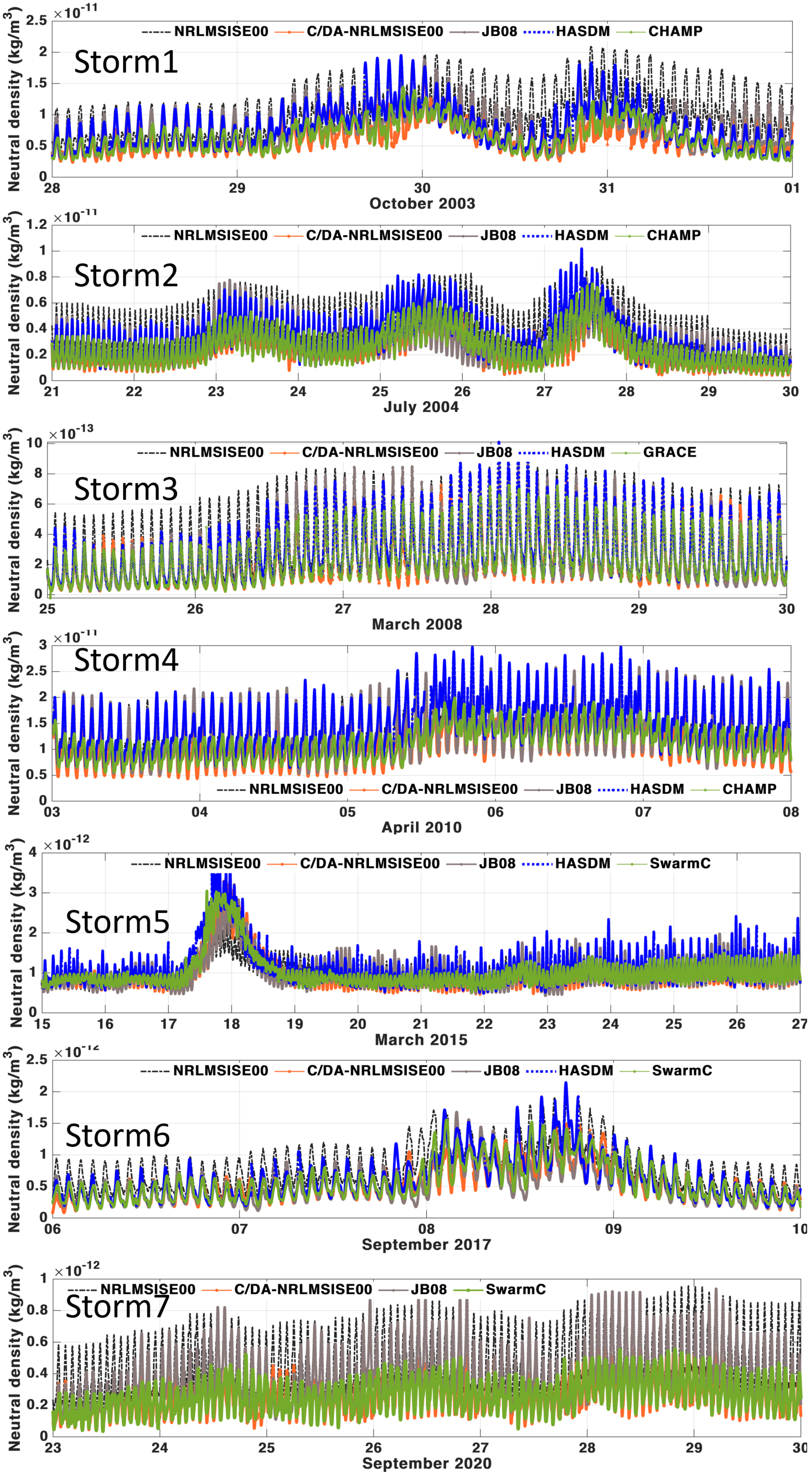

Figure 1. A comparison between the forecasts of C/DA-NRLMSISE-00 and those of the original NRLMSISE-00 model, as well as JB08, HASDM models along-track of the satellites that were used to estimate the global multi-level data. The C/DA
